# Supplementary material for: Phage vB_KlebPS_265 Active Against Resistant/MDR and Hypermucoid K2 Strains of Klebsiella pneumoniae
Source: Viruses. 2025 Jan 9;17(1):83. doi: 10.3390/v17010083 (PMC11769527; doi:10.3390/v17010083)
Supplement: Supplementary file 1 [file viruses-17-00083-s001.zip › Figure S1.pdf]

A

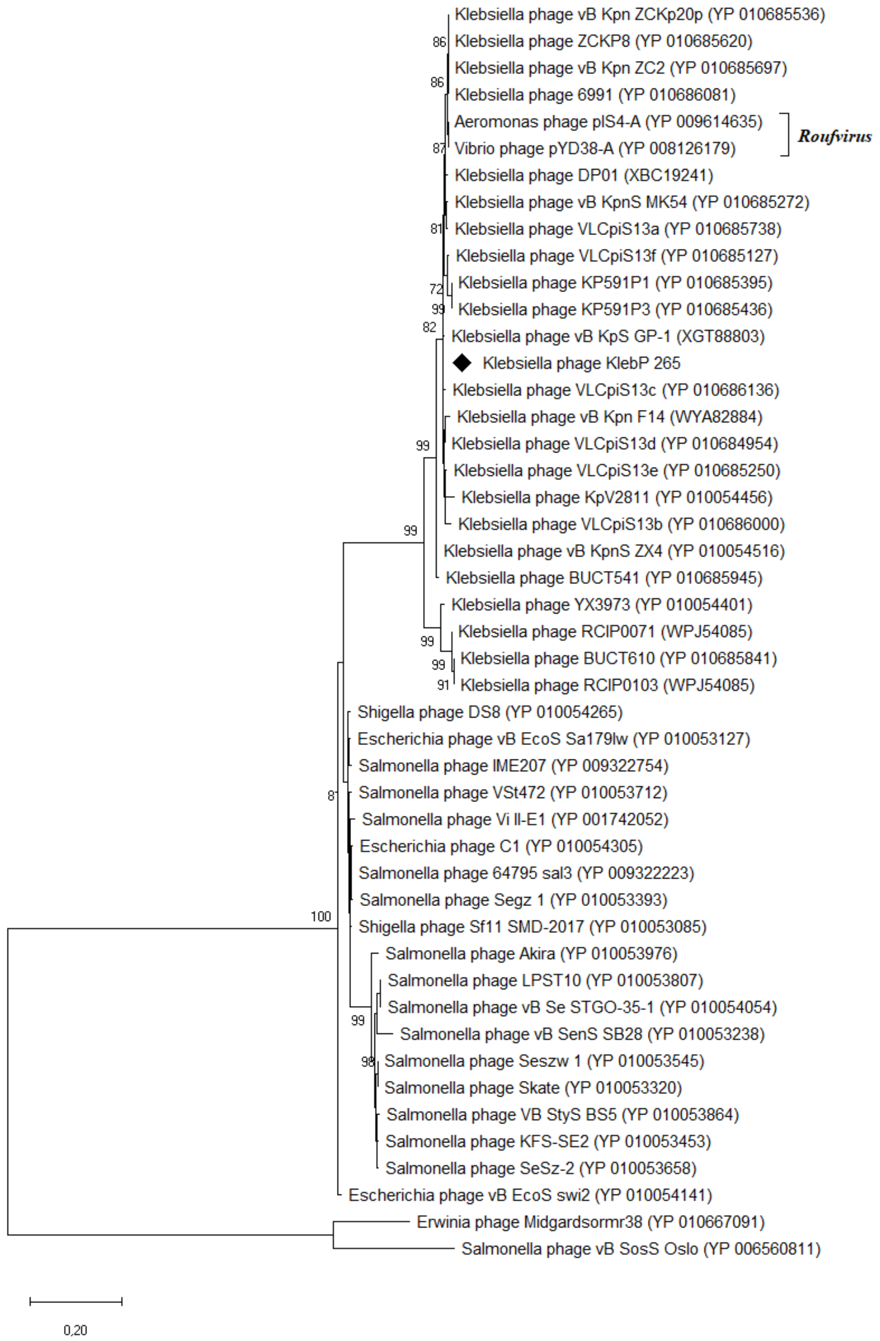
Klebsiella phage vB Kpn ZCKp20p (YP 010685536)
86
Klebsiella phage ZCKP8 (YP 010685620)
Klebsiella phage vB Kpn ZC2 (YP 010685697)
86
Klebsiella phage 6991 (YP 010686081)
Aeromonas phage pIS4-A (YP 009614635)
Roufvirus
87
Vibrio phage pYD38-A (YP 008126179)
Klebsiella phage DP01 (XBC19241)
Klebsiella phage vB KpnS MK54 (YP 010685272)
81
Klebsiella phage VLCpiS13a (YP 010685738)
Klebsiella phage VLCpiS13f (YP 010685127)
72
Klebsiella phage KP591P1 (YP 010685395)
99
Klebsiella phage KP591P3 (YP 010685436)
82
Klebsiella phage vB KpS GP-1 (XGT88803)
Klebsiella phage KlebP 265
Klebsiella phage VLCpiS13c (YP 010686136)
Klebsiella phage vB Kpn F14 (WYA82884)
99
Klebsiella phage VLCpiS13d (YP 010684954)
Klebsiella phage VLCpiS13e (YP 010685250)
Klebsiella phage KpV2811 (YP 010054456)
99
Klebsiella phage VLCpiS13b (YP 010686000)
Klebsiella phage vB KpnS ZX4 (YP 010054516)
Klebsiella phage BUCT541 (YP 010685945)
Klebsiella phage YX3973 (YP 010054401)
99
Klebsiella phage RCIP0071 (WPJ54085)
99
Klebsiella phage BUCT610 (YP 010685841)
91
Klebsiella phage RCIP0103 (WPJ54085)
Shigella phage DS8 (YP 010054265)
Escherichia phage vB EcoS Sa179lw (YP 010053127)
Salmonella phage IME207 (YP 009322754)
81
Salmonella phage VSt472 (YP 010053712)
Salmonella phage Vi II-E1 (YP 001742052)
Escherichia phage C1 (YP 010054305)
Salmonella phage 64795 sal3 (YP 009322223)
Salmonella phage Segz 1 (YP 010053393)
100
Shigella phage Sf11 SMD-2017 (YP 010053085)
Salmonella phage Akira (YP 010053976)
Salmonella phage LPST10 (YP 010053807)
Salmonella phage vB Se STGO-35-1 (YP 010054054)
99
Salmonella phage vB SenS SB28 (YP 010053238)
98
Salmonella phage Seszw 1 (YP 010053545)
Salmonella phage Skate (YP 010053320)
Salmonella phage VB StyS BS5 (YP 010053864)
Salmonella phage KFS-SE2 (YP 010053453)
Salmonella phage SeSz-2 (YP 010053658)
Escherichia phage vB EcoS swi2 (YP 010054141)
Erwinia phage Midgardsormr38 (YP 010667091)
Salmonella phage vB SosS Oslo (YP 006560811)
0,20

B

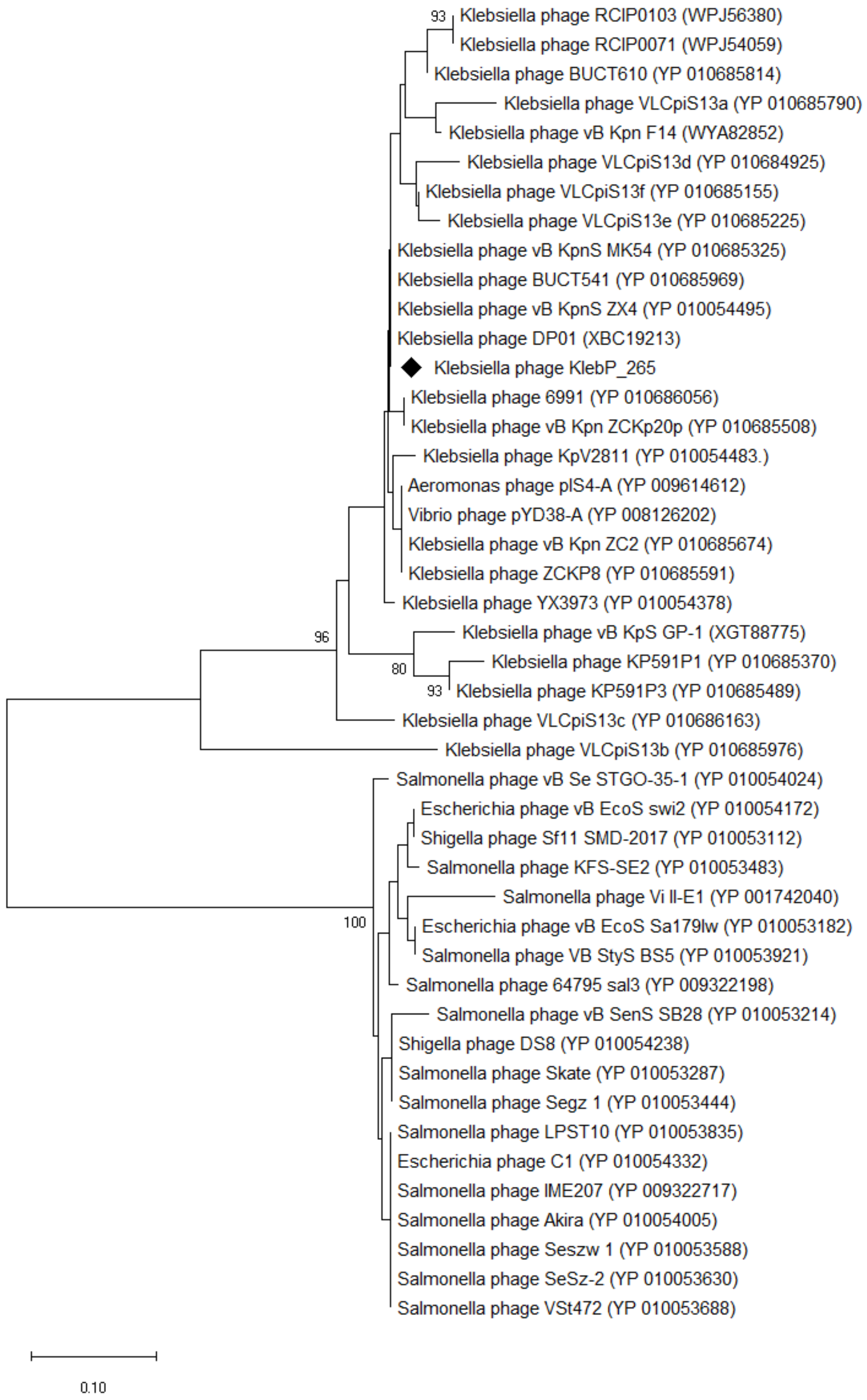

93
Klebsiella phage RCIP0103 (WPJ56380)
Klebsiella phage RCIP0071 (WPJ54059)
Klebsiella phage BUCT610 (YP 010685814)
Klebsiella phage VLCpiS13a (YP 010685790)
Klebsiella phage vB Kpn F14 (WYA82852)
Klebsiella phage VLCpiS13d (YP 010684925)
Klebsiella phage VLCpiS13f (YP 010685155)
Klebsiella phage VLCpiS13e (YP 010685225)
Klebsiella phage vB KpnS MK54 (YP 010685325)
Klebsiella phage BUCT541 (YP 010685969)
Klebsiella phage vB KpnS ZX4 (YP 010054495)
Klebsiella phage DP01 (XBC19213)
Klebsiella phage KlebP_265
Klebsiella phage 6991 (YP 010686056)
Klebsiella phage vB Kpn ZCKp20p (YP 010685508)
Klebsiella phage KpV2811 (YP 010054483.)
Aeromonas phage pIS4-A (YP 009614612)
Vibrio phage pYD38-A (YP 008126202)
Klebsiella phage vB Kpn ZC2 (YP 010685674)
Klebsiella phage ZCKP8 (YP 010685591)
Klebsiella phage YX3973 (YP 010054378)
96
Klebsiella phage vB KpS GP-1 (XGT88775)
80
Klebsiella phage KP591P1 (YP 010685370)
93
Klebsiella phage KP591P3 (YP 010685489)
Klebsiella phage VLCpiS13c (YP 010686163)
Klebsiella phage VLCpiS13b (YP 010685976)
Salmonella phage vB Se STGO-35-1 (YP 010054024)
Escherichia phage vB EcoS swi2 (YP 010054172)
Shigella phage Sf11 SMD-2017 (YP 010053112)
Salmonella phage KFS-SE2 (YP 010053483)
Salmonella phage Vi II-E1 (YP 001742040)
Escherichia phage vB EcoS Sa179lw (YP 010053182)
Salmonella phage VB StyS BS5 (YP 010053921)
100
Salmonella phage 64795 sal3 (YP 009322198)
Salmonella phage vB SenS SB28 (YP 010053214)
Shigella phage DS8 (YP 010054238)
Salmonella phage Skate (YP 010053287)
Salmonella phage Segz 1 (YP 010053444)
Salmonella phage LPST10 (YP 010053835)
Escherichia phage C1 (YP 010054332)
Salmonella phage IME207 (YP 009322717)
Salmonella phage Akira (YP 010054005)
Salmonella phage Seszw 1 (YP 010053588)
Salmonella phage SeSz-2 (YP 010053630)
Salmonella phage VSt472 (YP 010053688)
0.10

C

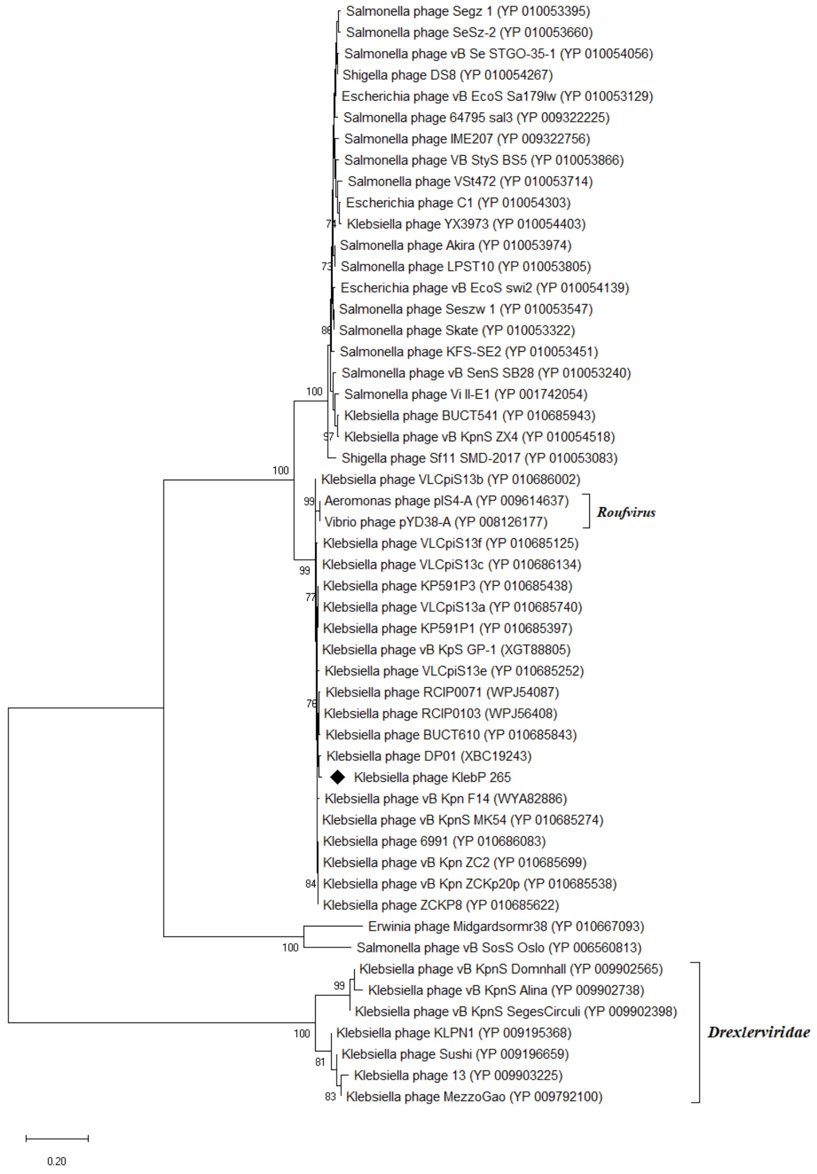
Salmonella phage Segz 1 (YP 010053395)
Salmonella phage SeSz-2 (YP 010053660)
Salmonella phage vB Se STGO-35-1 (YP 010054056)
Shigella phage DS8 (YP 010054267)
Escherichia phage vB EcoS Sa179lw (YP 010053129)
Salmonella phage 64795 sal3 (YP 009322225)
Salmonella phage IME207 (YP 009322756)
Salmonella phage VB StyS BS5 (YP 010053866)
Salmonella phage VSt472 (YP 010053714)
Escherichia phage C1 (YP 010054303)
Klebsiella phage YX3973 (YP 010054403)
Salmonella phage Akira (YP 010053974)
Salmonella phage LPST10 (YP 010053805)
Escherichia phage vB EcoS swi2 (YP 010054139)
Salmonella phage Seszw 1 (YP 010053547)
Salmonella phage Skate (YP 010053322)
Salmonella phage KFS-SE2 (YP 010053451)
Salmonella phage vB SenS SB28 (YP 010053240)
Salmonella phage Vi II-E1 (YP 001742054)
Klebsiella phage BUCT541 (YP 010685943)
Klebsiella phage vB KpnS ZX4 (YP 010054518)
Shigella phage Sf11 SMD-2017 (YP 010053083)
Klebsiella phage VLCpiS13b (YP 010686002)
Aeromonas phage pIS4-A (YP 009614637)
Vibrio phage pYD38-A (YP 008126177)
Roufvirus
Klebsiella phage VLCpiS13f (YP 010685125)
Klebsiella phage VLCpiS13c (YP 010686134)
Klebsiella phage KP591P3 (YP 010685438)
Klebsiella phage VLCpiS13a (YP 010685740)
Klebsiella phage KP591P1 (YP 010685397)
Klebsiella phage vB KpS GP-1 (XGT88805)
Klebsiella phage VLCpiS13e (YP 010685252)
Klebsiella phage RCIP0071 (WPJ54087)
Klebsiella phage RCIP0103 (WPJ56408)
Klebsiella phage BUCT610 (YP 010685843)
Klebsiella phage DP01 (XBC19243)
Klebsiella phage KlebP 265
Klebsiella phage vB Kpn F14 (WYA82886)
Klebsiella phage vB KpnS MK54 (YP 010685274)
Klebsiella phage 6991 (YP 010686083)
Klebsiella phage vB Kpn ZC2 (YP 010685699)
Klebsiella phage vB Kpn ZCKp20p (YP 010685538)
Klebsiella phage ZCKP8 (YP 010685622)
Erwinia phage Midgardsormr38 (YP 010667093)
Salmonella phage vB SosS Oslo (YP 006560813)
Klebsiella phage vB KpnS Domnhall (YP 009902565)
Klebsiella phage vB KpnS Alina (YP 009902738)
Klebsiella phage vB KpnS SegesCirculi (YP 009902398)
Klebsiella phage KLPN1 (YP 009195368)
Klebsiella phage Sushi (YP 009196659)
Klebsiella phage 13 (YP 009903225)
Klebsiella phage MezzoGao (YP 009792100)
Drexlerviridae
100
100
99
99
77
76
84
100
99
100
81
83
0.20

D

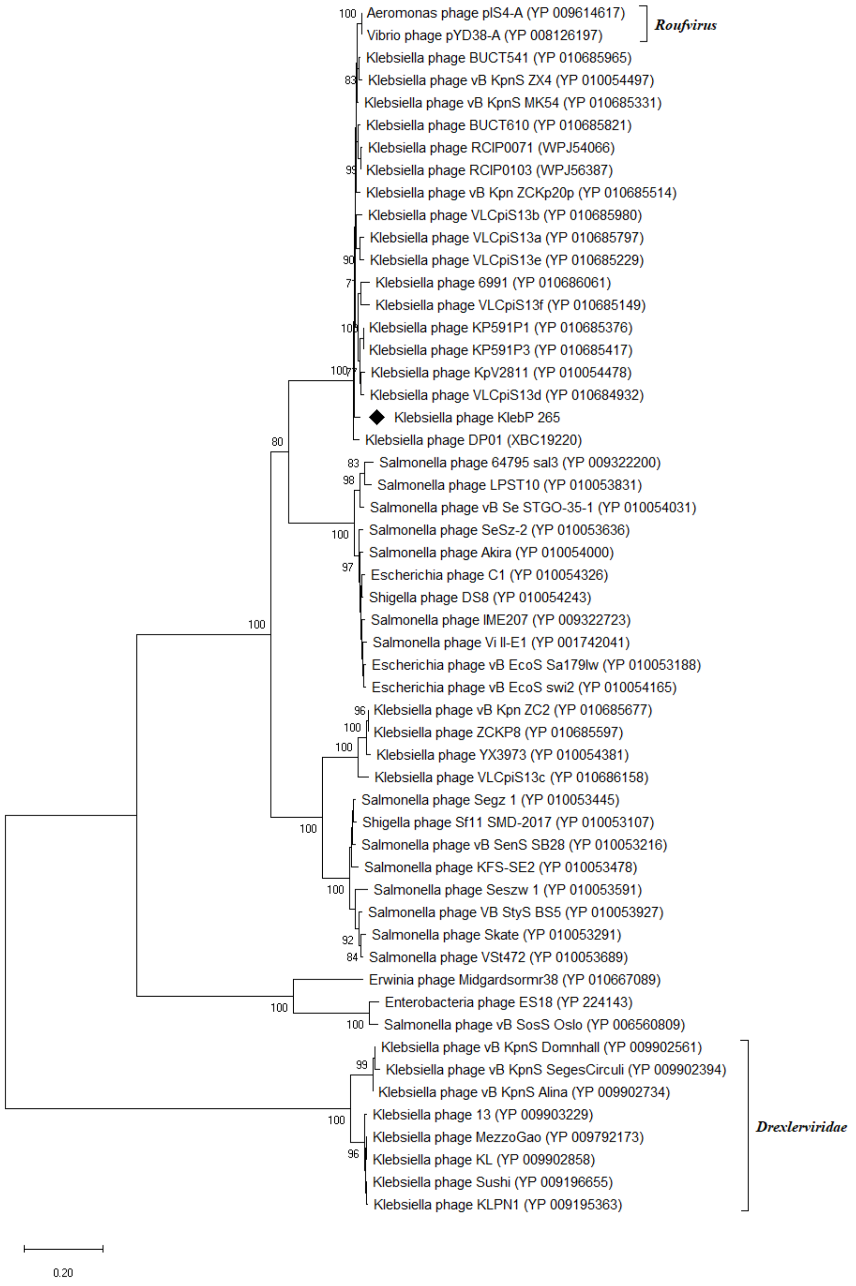
Aeromonas phage pIS4-A (YP 009614617)
Vibrio phage pYD38-A (YP 008126197)
Roufvirus
Klebsiella phage BUCT541 (YP 010685965)
Klebsiella phage vB KpnS ZX4 (YP 010054497)
Klebsiella phage vB KpnS MK54 (YP 010685331)
Klebsiella phage BUCT610 (YP 010685821)
Klebsiella phage RCIP0071 (WPJ54066)
Klebsiella phage RCIP0103 (WPJ56387)
Klebsiella phage vB Kpn ZCKp20p (YP 010685514)
Klebsiella phage VLCpiS13b (YP 010685980)
Klebsiella phage VLCpiS13a (YP 010685797)
Klebsiella phage VLCpiS13e (YP 010685229)
Klebsiella phage 6991 (YP 010686061)
Klebsiella phage VLCpiS13f (YP 010685149)
Klebsiella phage KP591P1 (YP 010685376)
Klebsiella phage KP591P3 (YP 010685417)
Klebsiella phage KpV2811 (YP 010054478)
Klebsiella phage VLCpiS13d (YP 010684932)
Klebsiella phage KlebP 265
Klebsiella phage DP01 (XBC19220)
Salmonella phage 64795 sal3 (YP 009322200)
Salmonella phage LPST10 (YP 010053831)
Salmonella phage vB Se STGO-35-1 (YP 010054031)
Salmonella phage SeSz-2 (YP 010053636)
Salmonella phage Akira (YP 010054000)
Escherichia phage C1 (YP 010054326)
Shigella phage DS8 (YP 010054243)
Salmonella phage IME207 (YP 009322723)
Salmonella phage Vi II-E1 (YP 001742041)
Escherichia phage vB EcoS Sa179lw (YP 010053188)
Escherichia phage vB EcoS swi2 (YP 010054165)
Klebsiella phage vB Kpn ZC2 (YP 010685677)
Klebsiella phage ZCKP8 (YP 010685597)
Klebsiella phage YX3973 (YP 010054381)
Klebsiella phage VLCpiS13c (YP 010686158)
Salmonella phage Segz 1 (YP 010053445)
Shigella phage Sf11 SMD-2017 (YP 010053107)
Salmonella phage vB SenS SB28 (YP 010053216)
Salmonella phage KFS-SE2 (YP 010053478)
Salmonella phage Seszw 1 (YP 010053591)
Salmonella phage VB StyS BS5 (YP 010053927)
Salmonella phage Skate (YP 010053291)
Salmonella phage VSt472 (YP 010053689)
Erwinia phage Midgardsormr38 (YP 010667089)
Enterobacteria phage ES18 (YP 224143)
Salmonella phage vB SosS Oslo (YP 006560809)
Klebsiella phage vB KpnS Domnhall (YP 009902561)
Klebsiella phage vB KpnS SegesCirculi (YP 009902394)
Klebsiella phage vB KpnS Alina (YP 009902734)
Klebsiella phage 13 (YP 009903229)
Klebsiella phage MezzoGao (YP 009792173)
Klebsiella phage KL (YP 009902858)
Klebsiella phage Sushi (YP 009196655)
Klebsiella phage KLPN1 (YP 009195363)
Drexlerviridae
0.20

E

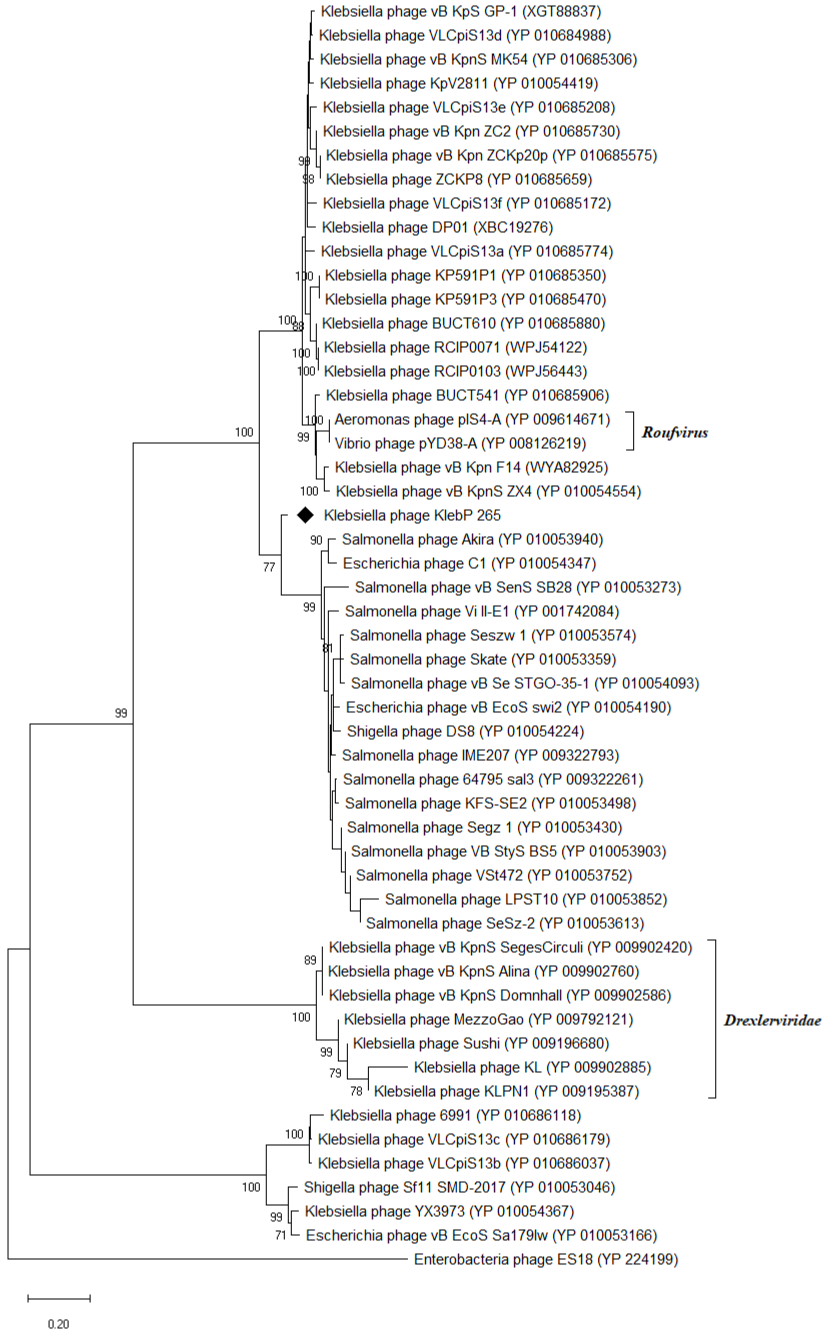
Klebsiella phage vB KpS GP-1 (XGT88837)
Klebsiella phage VLCpiS13d (YP 010684988)
Klebsiella phage vB KpnS MK54 (YP 010685306)
Klebsiella phage KpV2811 (YP 010054419)
Klebsiella phage VLCpiS13e (YP 010685208)
Klebsiella phage vB Kpn ZC2 (YP 010687730)
Klebsiella phage vB Kpn ZCKp20p (YP 010685575)
Klebsiella phage ZCKP8 (YP 010685659)
Klebsiella phage VLCpiS13f (YP 010685172)
Klebsiella phage DP01 (XBC19276)
Klebsiella phage VLCpiS13a (YP 010685774)
Klebsiella phage KP591P1 (YP 010685350)
Klebsiella phage KP591P3 (YP 010685470)
Klebsiella phage BUCT610 (YP 010685880)
Klebsiella phage RCIP0071 (WPJ54122)
Klebsiella phage RCIP0103 (WPJ56443)
Klebsiella phage BUCT541 (YP 010685906)
Aeromonas phage pIS4-A (YP 009614671)
Vibrio phage pYD38-A (YP 008126219)
Roufvirus
Klebsiella phage vB Kpn F14 (WYA82925)
Klebsiella phage vB KpnS ZX4 (YP 010054554)
Klebsiella phage KlebP 265
Salmonella phage Akira (YP 010053940)
Escherichia phage C1 (YP 010054347)
Salmonella phage vB SenS SB28 (YP 010053273)
Salmonella phage Vi II-E1 (YP 001742084)
Salmonella phage Seszw 1 (YP 010053574)
Salmonella phage Skate (YP 010053359)
Salmonella phage vB Se STGO-35-1 (YP 010054093)
Escherichia phage vB EcoS swi2 (YP 010054190)
Shigella phage DS8 (YP 010054224)
Salmonella phage IME207 (YP 009322793)
Salmonella phage 64795 sal3 (YP 009322261)
Salmonella phage KFS-SE2 (YP 010053498)
Salmonella phage Segz 1 (YP 010053430)
Salmonella phage VB StyS BS5 (YP 010053903)
Salmonella phage VSt472 (YP 010053752)
Salmonella phage LPST10 (YP 010053852)
Salmonella phage SeSz-2 (YP 010053613)
Klebsiella phage vB KpnS SegesCirculi (YP 009902420)
Klebsiella phage vB KpnS Alina (YP 009902760)
Klebsiella phage vB KpnS Domnhall (YP 009902586)
Klebsiella phage MezzoGao (YP 009792121)
Klebsiella phage Sushi (YP 009196680)
Klebsiella phage KL (YP 009902885)
Klebsiella phage KLPN1 (YP 009195387)
Drexlerviridae
Klebsiella phage 6991 (YP 010686118)
Klebsiella phage VLCpiS13c (YP 010686179)
Klebsiella phage VLCpiS13b (YP 010686037)
Shigella phage Sf11 SMD-2017 (YP 010053046)
Klebsiella phage YX3973 (YP 010054367)
Escherichia phage vB EcoS Sa179lw (YP 010053166)
Enterobacteria phage ES18 (YP 224199)
0.20

F

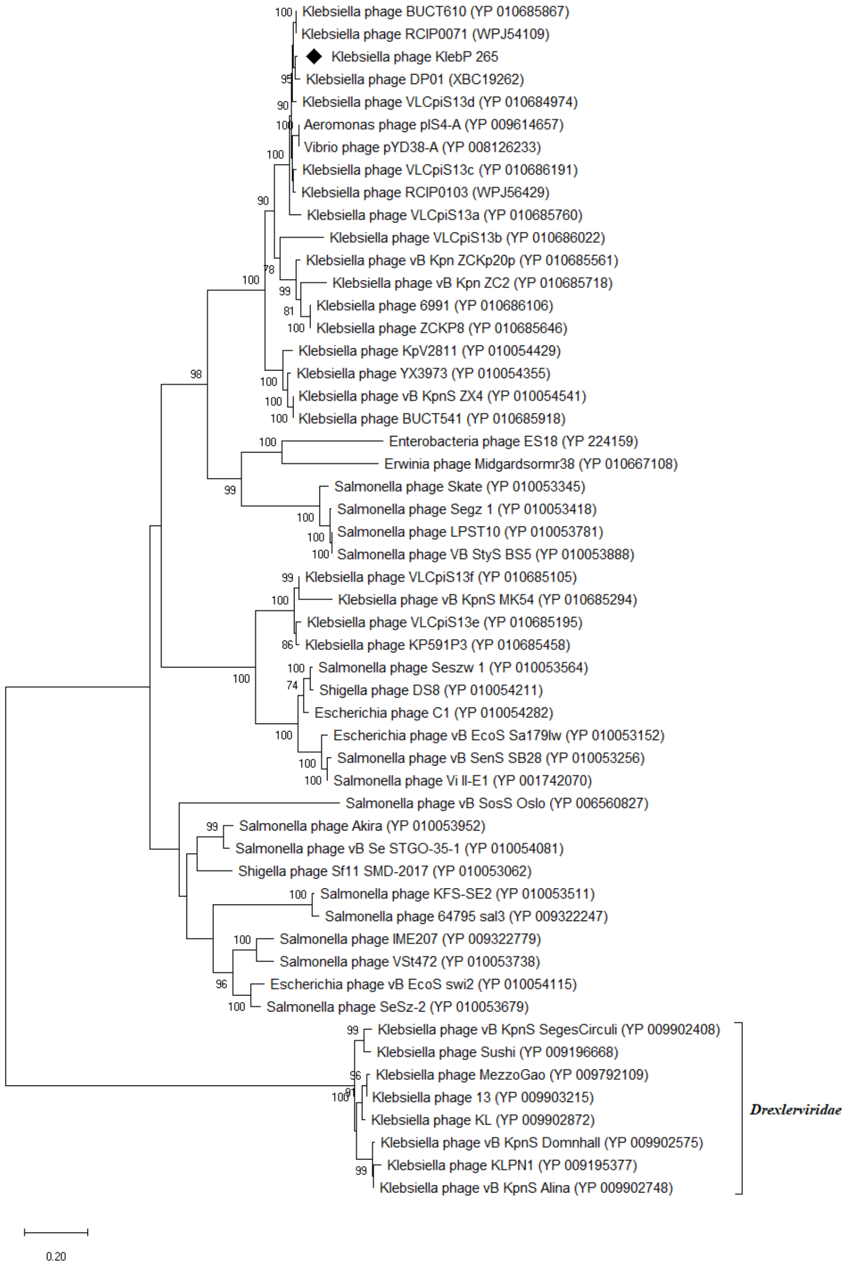

Figure S1. Phylogenetic analysis of the essential proteins of the phage KlebP_265: (A) prohead core protease; (B) InsA C-terminal domain protein; (C) major capsid protein; (D) portal protein; (E) DNA primase; (F) tail length tape measure protein. Sequences were aligned using CLUSTALW, phylogenetic tree constructed using MEGA 11.0. Maximum-Likelyhood method with bootstrap 1000 was applied. Protein sequences of phage KlebP_265 are marked with black diamonds.
