# Supplementary figures and images for: Phage vB_KlebPS_265 Active Against Resistant/MDR and Hypermucoid K2 Strains of Klebsiella pneumoniae
Source: Viruses. 2025 Jan 9;17(1):83. doi: 10.3390/v17010083 (PMC11769527; doi:10.3390/v17010083)

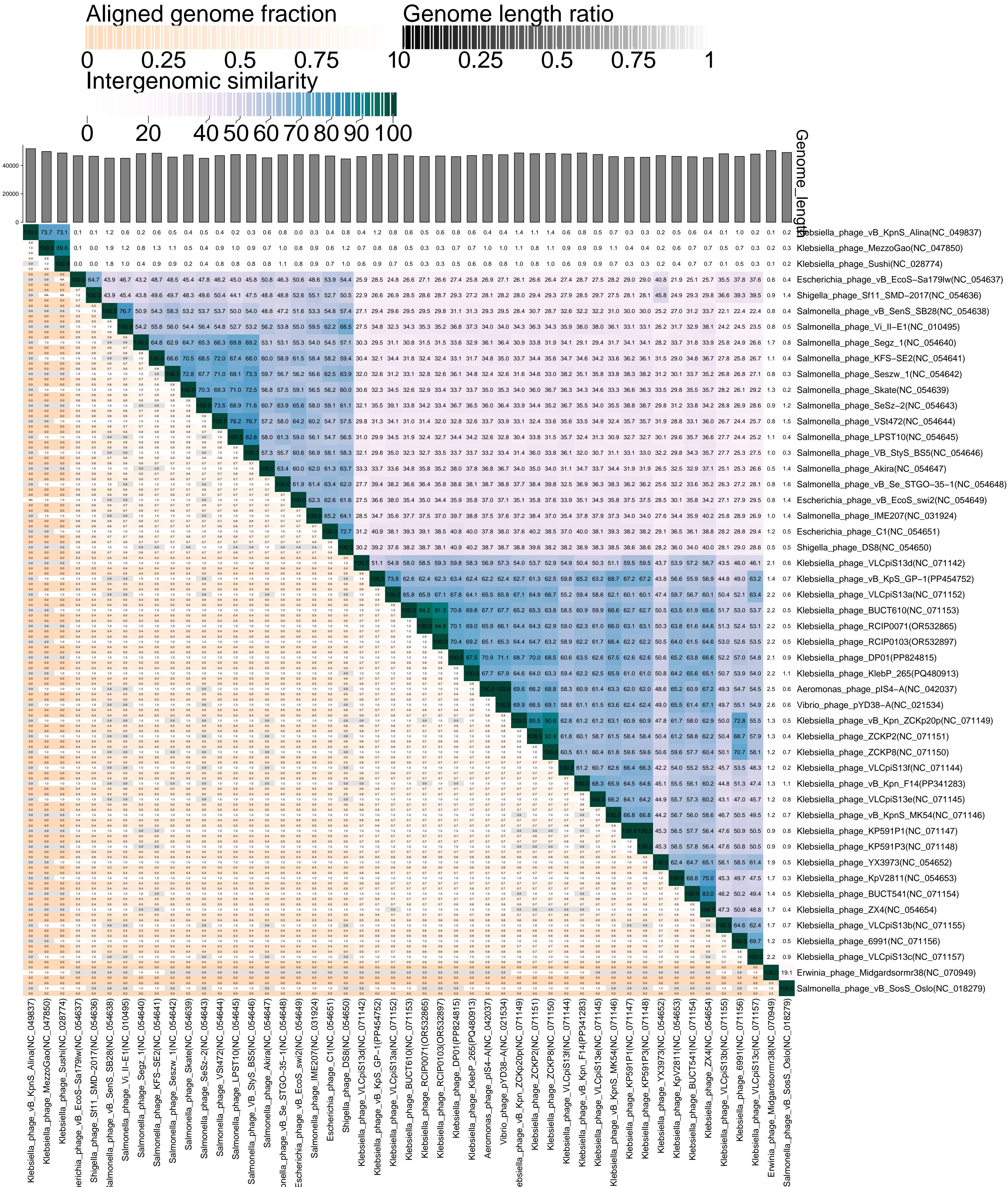

Supplement: Supplementary file 1 [file viruses-17-00083-s001.zip › Data S2.pdf]
